# Reduced expression of PPAR-β/δ limits the potential beneficial effects of GW0742 during septic shock in atherosclerotic swine

**DOI:** 10.1186/cc10625

**Published:** 2012-03-20

**Authors:** H Bracht, F Simon, J Matallo, M Gröger, O McCook, A Seifritz, M Georgieff, E Calzia, P Radermacher, A Kapoor, C Thiemermann

**Affiliations:** 1 University Clinic Ulm, Germany; 2 William Harvey Research Institute, London, UK

## Introduction

The PPAR-β/δ agonist GW0742 was shown to attenuate cardiac dysfunction in murine septic shock [1] and renal ischemia/reperfusion injury in diabetic rats [2]. Since these data originate from unresuscitated models, we investigated the effects of GW0742 during long-term, resuscitated porcine septic shock. In order to assess the role of pre-existing cardiovascular morbidity we used animals with familial hypercholesteremia (11.1 (7.4; 12.3) vs. 1.4 (1.3; 1.5) mmol/l in a healthy strain; *P *< 0.001) and consecutive, diet-induced ubiquitous atherosclerosis resulting in coronary artery disease [3], reduced glomerular filtration rate (76 (60; 83) vs. 103 (79; 120) ml/minute in healthy swine; *P *= 0.004) and presence of chronic histological kidney injury.

## Methods

Anesthetized and instrumented animals randomly received vehicle (*n *= 9) or GW0742 (*n *= 10; 0.03 mg/kg) at 6, 12, 18 hours after induction of fecal peritonitis [4]. Hydroxyethyl starch and noradrenaline were infused to maintain normotensive, hyperdynamic hemodynamics. Creatinine clearance was measured from 0 to 12 hours and from 12 to 24 hours of sepsis, respectively. Data are median (quartiles).

## Results

GW0742 did not affect the noradrenaline infusion rate required to achieve target hemodynamics (0.57 (0.30; 3.83) vs. 0.56 (0.41; 0.91) μg/kg/minute; *P *= 0.775) nor the fall in creatinine clearance (GW0742: from 129 (114; 140) to 78 (55; 95) ml/minute, *P *= 0.002; vehicle: from 130 (91; 142) to 41(31; 84) ml/minute, *P *= 0.004; *P *= 0.967 and *P *= 0.191 between groups). Immune histochemistry analysis of kidney biopsies in sham-operated swine showed markedly reduced tissue expression of the PPAR-β/δ receptor in atherosclerotic swine (281 (277; 404) vs. 57 (53; 77)×10^3 ^densitometric units in healthy swine; *P *= 0.008).

## Conclusion

Even early post-treatment with the PPAR-β/δ agonist GW0742 did not beneficially influence acute kidney injury during long-term, resuscitated fecal peritonitis-induced septic shock in swine with pre-existing impairment of kidney function and histological damage. The lacking beneficial effect of GW0742 was most likely due to the reduced expression of the PPAR-β/δ receptor.
